# Joint Association of Functional Impairment and Depression with Advanced Cardiovascular–Kidney–Metabolic Syndrome: A Cross-Sectional and Cohort Study Using Two Large Datasets

**DOI:** 10.3390/healthcare14142043

**Published:** 2026-07-08

**Authors:** Yaning Zheng, Ruifeng Wang

**Affiliations:** 1 Department of Nephrology, Second Affiliated Hospital of Anhui Medical University, Hefei 230601, China; 2 Department of Nephrology, Xianning Central Hospital, First Affiliated Hospital of Hubei University of Science and Technology, Xianning 437100, China

**Keywords:** cardiovascular–kidney–metabolic syndrome, activities of daily living, functional impairment, depression

## Abstract

**Background:** Unfavorable cardiovascular–kidney–metabolic (CKM) status represents a major contributor to all-cause deaths. Our study sought to investigate the joint association of functional impairment and depression with advanced CKM syndrome among individuals of middle and old age. **Methods:** Participants for the cross-sectional analysis were drawn from the National Health and Nutrition Examination Survey (NHANES) in the United States, while those for the cohort analysis were selected from the China Health and Retirement Longitudinal Study (CHARLS). To analyze the independent and joint associations of functional impairment, depression, and their comorbidity with advanced CKM syndrome, multivariable logistic regression was performed. Subgroup analyses were performed to evaluate effect variations and interactions. **Results:** In the NHANES sample, the coexistence of instrumental activities of daily living (IADL)/basic activities of daily living (BADL) impairment and depression showed a significant positive association with advanced CKM syndrome (IADL and depression: odds ratio [OR] = 4.843, 95% confidence interval [CI]: 2.505, 9.366; BADL and depression: OR = 4.394, 95% CI: 2.281, 8.464); In the CHARLS cohort, the coexistence of IADL/BADL impairment and depression showed a stronger association with advanced CKM syndrome (IADL and depression: OR = 2.259, 95% CI: 1.517, 3.319; BADL and depression: OR = 2.287, 95% CI: 1.501, 3.419). **Conclusions:** The presence of functional impairment and depression is significantly associated with advanced CKM syndrome. Screening for coexisting functional impairment and depression may help identify individuals who are more likely to develop advanced CKM syndrome.

## 1. Introduction

Cardiovascular–kidney–metabolic (CKM) syndrome stems from the pathophysiological interactions between metabolic risk factors (e.g., obesity and diabetes) and chronic kidney disease (CKD) and cardiovascular disease (CVD) [1]. Recent research has indicated that CKM syndrome is extremely prevalent among American adults [2]. It should be noted that 15% of U.S. adults suffer from CKM syndrome of advanced stages (3 or 4) [3]. Advanced CKM syndrome can affect nearly all major organ systems throughout the body, leading to numerous difficulties in treatment and prognosis assessment, including end-stage kidney disease, serious metabolic disorders, and elevated predisposition to malignancies [4,5,6]. To delay the onset of advanced CKM syndrome, it is essential to establish a robust stratification system to assess the health condition of patients with CKM syndrome from multiple dimensions. This approach optimizes individualized treatment strategies and enables the rational allocation of medical resources.

Research has shown that many factors are closely linked to the onset and progression of advanced CKM syndrome. Sleep quality, physical activity levels, and the severity of depression are all closely linked to advanced CKM syndrome [7,8,9]. Although the aforementioned studies have explored multiple risk factors associated with advanced CKM syndrome, they have all focused on single factors related to functional status or mental health. Currently, the impact of combined indicators of functional status and mental health on advanced CKM syndrome remains unexplored.

With the rapid progression of population aging, functional impairment and depression among older individuals have become issues that cannot be overlooked in the pursuit of healthy aging. Functional impairment refers to the diminished capacity to perform activities of daily living (ADL) and intricate tasks required for living with independence [10]. Studies have shown that functional impairment leads to weight gain, obesity, and dyslipidemia, thereby accelerating the progression of CKM syndrome [11,12]. Depression represents the most prevalent mental disorder in primary care and mental health settings and constitutes a major global public health issue [13]. It has been reported that the global prevalence of depression ranges from 2% to 6% [14]. A previous study has demonstrated a bidirectional association between depression and chronic diseases, that is, depression may exacerbate the progression of physical illnesses, and vice versa [15]. Depression can induce systemic inflammatory responses through multiple pathways, and inflammation represents a key factor accelerating the progression of CKM syndrome [16,17]. Meanwhile, the co-occurrence of depression and functional impairment is common, and a close association between the two has been well demonstrated [18,19,20]. Both functional impairment and depression can drive the progression of CKM syndrome by impairing an individual’s self-management capabilities, including medication adherence and health-promoting behaviors. Based on this, investigating the joint association of functional impairment and depression with advanced CKM syndrome is of significant practical importance, yet this area remains unexplored.

Hence, our study sought to explore the individual and combined associations of functional impairment and depression with advanced CKM syndrome, leveraging data from two national population-based databases: the National Health and Nutrition Examination Survey (NHANES) and the China Health and Retirement Longitudinal Study (CHARLS). Through integrating complementary evidence from these two large databases—one providing cross-sectional data from the U.S. and the other offering longitudinal data from China—this study was expected to more thoroughly clarify the association of functional impairment and depression with advanced CKM syndrome, thereby providing new insights for risk stratification and comprehensive interventions for patients with advanced CKM syndrome.

## 2. Methods

### 2.1. Study Population

**Figure 1:**
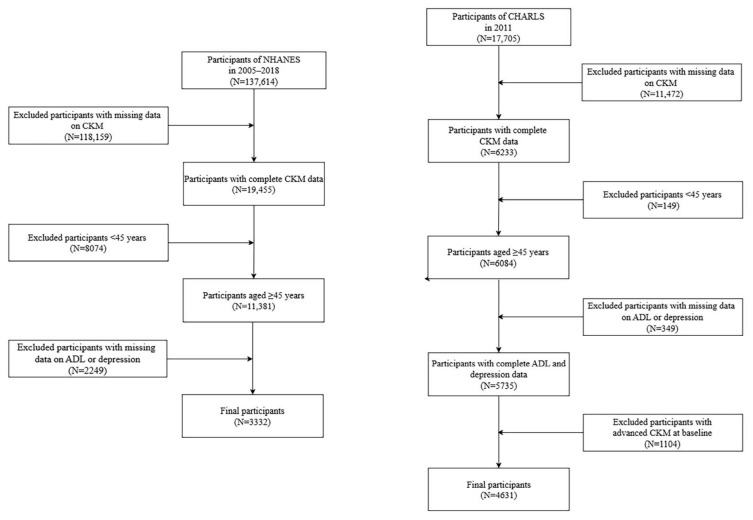
Flowchart of the study population.

NHANES is a large-scale, nationally representative, long-term, continuous cross-sectional survey led by the National Center for Health Statistics of the U.S. Centers for Disease Control and Prevention. The survey consistently adopts a multistage stratified sampling technique to collect nationally representative data on nutrition and health among non-hospitalized adults and children in the U.S. every two years [21]. Before data collection, all participants or their legal guardians granted written informed consent. Since our study involved secondary analysis of de-identified datasets, the risk to participants’ privacy is minimal. Therefore, ethical review was exempted. This study extracted and analyzed data from 7 consecutive survey cycles between 2005 and 2018. The exclusion criteria were as follows (Figure 1): (i) Participants with missing data on the definition of CKM syndrome (N = 118,103); (ii) Participants aged <45 years (N = 8114); (iii) Participants with missing data on the staging diagnosis of CKM syndrome (N = 5816); (iv) Participants with missing data on ADL or depression (N = 2249). Ultimately, 3332 participants from NHANES were included in this study.

CHARLS is a large-scale, interdisciplinary survey project led by the National School of Development of Peking University and undertaken by the Institute of Social Science Survey. It adopts a multistage stratified probability sampling technique to conduct continuous, periodic follow-up surveys on the same cohort of respondents [22]. Its sample came from 450 communities (villages) of 150 counties across 28 provincial regions, ensuring high representativeness of the survey at both the national and provincial levels. The baseline survey was started in 2011, followed by nationwide follow-up surveys every 2 years. The ethical approval for research protocol was granted by the Institutional Review Board of Peking University. All procedures were carried out following the principles of the Declaration of Helsinki, and written informed consent was obtained from all participants. This study employed a prospective cohort design with the first survey (2011) as the baseline and assessed follow-up outcomes through the third survey (2015). The following participants were excluded (Figure 1): (i) Participants with missing data on the definition of CKM syndrome (N = 7878); (ii) Participants aged <45 years (N = 251); (iii) Participants with missing data on the staging diagnosis of CKM syndrome (N = 3492); (iv) Participants with missing data on ADL or depression (N = 349); (v) Participants with advanced CKM syndrome at baseline (N = 1104). Ultimately, 4631 participants from CHARLS were included in this study.

### 2.2. Definitions of Variables

#### 2.2.1. Assessment of Functional Impairment

The assessment of functional impairment included basic activities of daily living (BADL) and instrumental activities of daily living (IADL). BADL refers to an individual’s core self-care abilities [23], whereas IADL refers to the organized, complex activities necessary for independent living in the community [24]. In NHANES, BADL assessment was based on 4 daily activities: walking between rooms on a single floor, getting in and out of bed, eating, and getting dressed, while IADL assessment covered 3 basic activities: managing finances, doing housework, and preparing meals. Respondents were queried regarding the extent of difficulty they encountered when executing the aforementioned tasks, with options including “no difficulty,” “some difficulty,” “great difficulty,” and “unable to perform.” Respondents who reported difficulty in ≥1 activity were classified as having an ADL impairment [25]. In CHARLS, BADL assessment comprises six items measuring getting dressed, taking a bath, eating, getting out of bed, using the toilet, and managing continence, while IADL assessment comprises five items measuring housekeeping, cooking, making phone calls, shopping, taking medicine, and managing finances. Each item was offered 4 options of response: (i) No difficulty; (ii) Difficulty but able to complete; (iii) Some difficulty, requiring assistance; (iv) Unable to complete. ADL impairment was defined as failure to complete any ADL item with full independence [26]. The ADL measures used in NHANES and CHARLS have been validated in prior studies conducted within their respective populations [27,28].

#### 2.2.2. Depression Assessment

In NHANES, the Patient Health Questionnaire-9 (PHQ-9) served as the assessment tool for depression. This 9-item self-report questionnaire was developed to assess and diagnose depression. A 0–3 rating scale was applied to each item, with the sum of all items ranging from 0 to 27. Participants with a PHQ-9 score of ≥10 were diagnosed with clinical depression [29]. The PHQ-9 has demonstrated a sensitivity of 88% and a specificity of 88% for detecting major depression at a cutoff of ≥10 in a large validation study [30]. In CHARLS, the Chinese version of the Center for Epidemiological Studies Depression (CES-D-10) scale served as the depression assessment tool. The scale yielded a total score between 0 and 30, where higher scores correspond to a greater severity of depression. Participants with a CES-D-10 score of ≥10 were diagnosed with clinical depression [31]. The CES-D-10 has shown satisfactory reliability and validity in older Chinese adults, with good internal consistency (Cronbach’s α ≥ 0.78) and strong concordance with the full CES-D scale [32].

The study classified participants with the following combinations of depression and ADL impairment: participants with no IADL/BADL impairment and no depression, participants with no IADL/BADL impairment but with depression, participants with IADL/BADL impairment but no depression, and participants with both IADL/BADL impairment and depression.

#### 2.2.3. Definitions of CKM Syndrome Stages 0–4

CKM syndrome was stratified as recommended by the American Heart Association, with staging ranging from 0 (no CKM risk factors) to 4 (the highest-risk stage in individuals diagnosed with CVD) [33]. The criteria for each stage were defined below. Stage 0 was defined as body mass index (BMI) below the ethnicity-specific threshold and waist circumference below the ethnicity- and sex-specific threshold. Stage 1 was defined as excess adiposity or prediabetes. Stage 2 was defined by the presence of metabolic risk factors, including obesity, type 2 diabetes, hypertension, hypertriglyceridemia, or moderately decreased estimated glomerular filtration rate (eGFR). Stage 3 was defined as severely decreased eGFR or high 10-year cardiovascular risk. Stage 4 was defined as established cardiovascular disease. The specific definitions for each stage are provided in Supplementary Table S1. A hierarchical classification was applied, with participants assigned to the highest applicable stage. In this study, only CKM syndrome at advanced stages (3–4) was included as the outcome.

### 2.3. Covariables

Based on previous literature and specialized knowledge [34,35], this study analyzed covariables including sex, age, educational attainment, marital status, BMI, smoking status, drinking status, hypertension, diabetes, dyslipidemia, and total sleep time. Sex was categorized as male or female. Educational attainment was categorized as less than high school, high school, or more than high school. Marital status was categorized as married/cohabiting, widowed/divorced/separated, or never married. Smoking and drinking status were categorized as “yes” or “no” in NHANES and as “current,” “former,” or “never” in CHARLS. Total sleep time was categorized as <7 h or ≥7 h. Hypertension was identified by any of the following: (i) self-reported history of hypertension diagnosed by a physician; (ii) average systolic pressure ≥ 140 mmHg or (and) average diastolic pressure ≥ 90 mmHg across three measurements; (iii) current use of antihypertensive agents. Diabetes was identified by any of the following: (i) self-reported history of diabetes diagnosed by a physician; (ii) glycated hemoglobin (HbA1c) ≥ 6.5%; (iii) fasting blood glucose ≥ 7.0 mmol/L; (iv) current use of antidiabetic agents; (v) current use of insulin. Dyslipidemia was identified by any of the following: (i) self-reported history of dyslipidemia diagnosed by a physician; (ii) low-density lipoprotein (LDL) cholesterol ≥ 130 mg/dL; (iii) high-density lipoprotein (HDL) cholesterol ≤ 40 mg/dL for men or ≤50 mg/dL for women; (iv) total cholesterol ≥ 200 mg/dL; (v) triglycerides ≥ 150 mg/dL; (vi) current use of lipid-lowering agents.

### 2.4. Statistical Analysis

Given that the missing rate of covariables in this study was less than 10%, random forest-based multiple imputation was employed to generate the complete dataset. Based on the complex multistage sampling in NHANES, all statistical analyses of the NHANES database integrated sampling weights, stratification, and primary sampling units to ensure nationally representative inferences. For continuous variables, the Shapiro–Wilk test was applied to evaluate normality. Normally distributed data were presented as mean ± standard deviation (SD), while non-normally distributed data were described as the median and interquartile range (IQR). For between-group comparisons of continuous variables, the t-test was used for normally distributed data, and the Mann–Whitney U test was employed for non-normally distributed data. Categorical variables were reported as frequency [percentage (%)], and compared between groups utilizing the chi-square test. To analyze the independent and joint associations of functional impairment, depression, and their comorbidity with advanced CKM syndrome, multivariable logistic regression was performed. Three models were established for comprehensive analysis. Model 1 was adjusted for age and sex. Model 2 additionally incorporated educational attainment and marital status based on Model 1. Model 3 additionally incorporated BMI, smoking status, drinking status, hypertension, diabetes, dyslipidemia, and total sleep time based on Model 2. In addition, moderation analysis was performed to examine whether depression or functional impairment moderated the association between the other variable and advanced CKM syndrome. Multicollinearity was evaluated utilizing the variance inflation factor (VIF), with a VIF >5 signifying the presence of multicollinearity [36].

Subgroup analyses were conducted to explore differences in the combined effect across different populations, and the likelihood ratio test was employed to assess interactions between the combined effect and different subgroups. Sensitivity analyses were performed via complete-case analysis, which excluded covariables with missing values, to test the robustness of the results. In addition, to address the concern that covariates such as BMI, hypertension, diabetes, and dyslipidemia may overadjust for components of CKM syndrome, another sensitivity analysis was conducted by excluding these variables. All statistical analyses were conducted in R 4.5.1, and statistical significance was identified by a two-sided *p*-value < 0.05.

## 3. Results

### 3.1. Baseline Population Characteristics

In strict accordance with the eligibility criteria, our study included 3332 participants from the NHANES database, with a median age of 66 years (61, 74) and a marginally higher percentage of female participants (52%) than male participants (48%). Among them, 1492 participants (45%) developed advanced CKM syndrome, who were predominantly male and older participants, with statistical significance (*p* < 0.05). In addition, participants with advanced CKM syndrome showed statistically significant differences from those without advanced CKM syndrome in marital status, educational attainment, BMI, smoking status, total sleep time, hypertension, diabetes, dyslipidemia, IADL impairment, BADL impairment, IADL impairment with concurrent depression, and BADL impairment with concurrent depression (*p* < 0.05) (Table 1).

Meanwhile, our study included 4631 participants from the CHARLS database, with a median age of 58 (52, 64) and a marginally higher percentage of female participants (55%) than male participants (45%). Among them, 247 participants (5%) developed new-onset advanced CKM syndrome, who were predominantly male and older participants, with statistical significance (*p* < 0.05). In addition, participants with advanced CKM syndrome showed statistically significant differences from those without advanced CKM syndrome in educational attainment, BMI, smoking status, total sleep time, hypertension, diabetes, dyslipidemia, depression, IADL impairment, BADL impairment, IADL impairment with concurrent depression, and BADL impairment with concurrent depression (*p* < 0.05) (Table 2).

### 3.2. Association of Functional Impairment and Depression with Advanced CKM Syndrome

Prior to logistic regression analysis, multicollinearity was assessed by calculating the VIF values for each variable. The results (Supplementary Tables S2 and S3) showed that all VIF values were <5, indicating no multicollinearity among the variables. Therefore, all variables could be incorporated into the model. In the NHANES sample, logistic regression results (Table 3) showed that participants with depression exhibited higher odds of developing advanced CKM syndrome than those without depression (OR = 3.930, 95% CI: 2.681, 5.760). After adjusting for all confounders (Model 3), the association between depression and advanced CKM syndrome persisted (OR = 3.306, 95% CI: 2.149, 5.086). Furthermore, after adjusting for all confounders (Model 3), participants with IADL or BADL impairment were more predisposed to advanced CKM syndrome than those without IADL or BADL (IADL: OR = 2.147, 95% CI: 1.520, 3.033; BADL: OR = 2.120, 95% CI: 1.565, 2.872). The combined analysis showed that, compared with the absence of IADL impairment and/or depression, the coexistence of IADL impairment and depression exhibited the strongest association with advanced CKM syndrome in both Model 1 and Model 3 (Model 1: OR = 6.294, 95% CI: 3.713, 10.670; Model 3: OR = 4.843, 95% CI: 2.505, 9.366). Compared with the absence of BADL impairment and/or depression, the coexistence of BADL impairment and depression exhibited the strongest association with advanced CKM syndrome in both Model 1 and Model 3 (Model 1: OR = 5.940, 95% CI: 3.357, 10.510; Model 3: OR = 4.394, 95% CI: 2.281, 8.464).

In the CHARLS cohort, logistic regression analysis (Table 4) showed that participants with depression exhibited higher odds of developing advanced CKM syndrome than those without depression (OR = 1.628, 95% CI: 1.252, 2.115). After adjusting for all confounders (Model 3), the association between depression and advanced CKM syndrome persisted (OR = 1.745, 95% CI: 1.324, 2.298). Furthermore, after adjusting for all confounders (Model 3), participants with IADL or BADL impairment were more predisposed to advanced CKM syndrome than those without IADL or BADL impairment (IADL: OR = 1.586, 95% CI: 1.147, 2.166; BADL: OR = 1.585, 95% CI: 1.124, 2.200). The combined analysis showed that, compared with participants without IADL impairment and/or depression, participants with both IADL impairment and depression exhibited the strongest association with advanced CKM syndrome in both Model 1 and Model 3 (Model 1: OR = 2.153, 95% CI: 1.470, 3.104; Model 3: OR = 2.259, 95% CI: 1.517, 3.319). Compared with participants without BADL impairment and/or depression, participants with both BADL impairment and depression exhibited the strongest association with advanced CKM syndrome in both Model 1 and Model 3 (Model 1: OR = 2.140, 95% CI: 1.428, 3.142; Model 3: OR = 2.287, 95% CI: 1.501, 3.419).

Moderation analysis revealed that depression moderated the association of IADL impairment and BADL impairment with advanced CKM syndrome in the NHANES dataset, but not in the CHARLS dataset (Supplementary Tables S4 and S5). Conversely, IADL impairment and BADL impairment moderated the association of depression with advanced CKM syndrome in the CHARLS dataset, but not in the NHANES dataset (Supplementary Tables S6 and S7).

### 3.3. Subgroup Analysis

**Figure 2:**
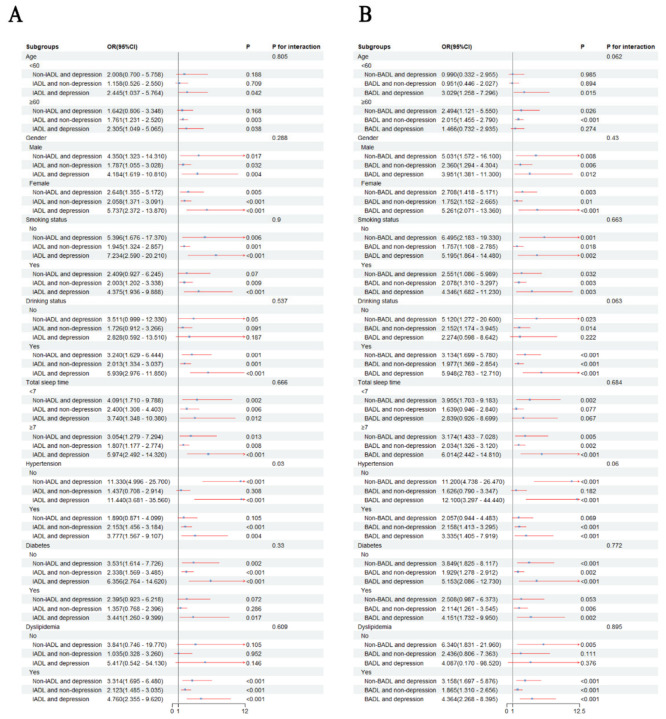
(**A**) Subgroup analysis of the joint association of IADL and depression with advanced CKM syndrome in NHANES; (**B**) subgroup analysis of the joint association of BADL and depression with advanced CKM syndrome in NHANES. The red line denote the 95% CI, and the blue square represent the OR. Abbreviations: OR: Odds ratio; CI: Confidence interval.

**Figure 3:**
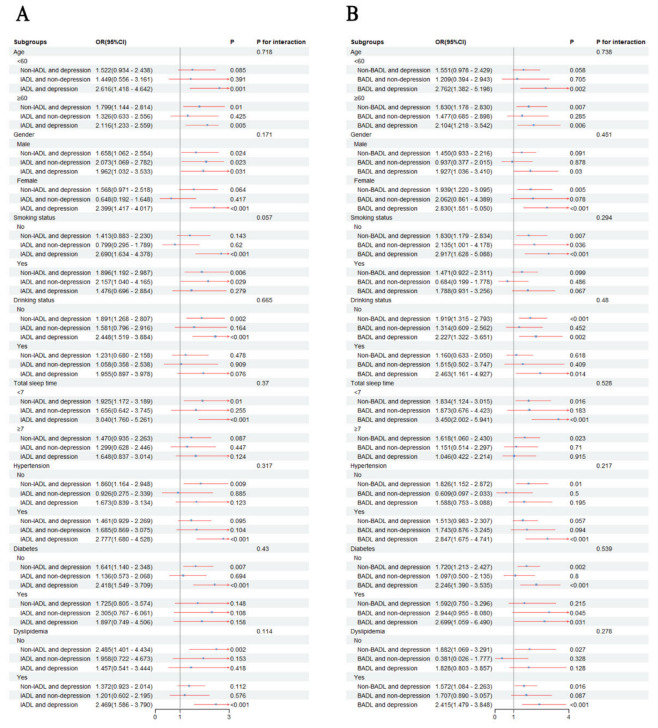
(**A**) Subgroup analysis of the joint association of IADL and depression with advanced CKM syndrome in CHARLS; (**B**) subgroup analysis of the joint association of BADL and depression with advanced CKM syndrome in CHARLS. The red line denote the 95% CI, and the blue square represent the OR. Abbreviations: OR: Odds ratio; CI: Confidence interval.

Subgroup analyses were conducted to evaluate potential effect modification across demo graphic and clinical variables, including age, sex, education, marital status, BMI, smoking, alcohol consumption, sleep time, hypertension, diabetes, and dyslipidemia. In the NHANES sample (Figure 2), only hypertension demonstrated a nominally significant interaction with the joint association of IADL impairment and depression (*p* = 0.03); however, this was not replicated in the CHARLS cohort (Figure 3). No other significant interactions were observed in either dataset (*p* > 0.05).

### 3.4. Sensitivity Analysis

Sensitivity analyses were performed via complete-case analysis, directly excluding covariables with missing values. The results (Supplementary Table S8) showed that, regardless of adjustment for covariables, the coexistence of depression and IADL or BADL impairment was associated with a greater predisposition to advanced CKM syndrome than the absence of depression and IADL or BADL impairment in both datasets. In addition, sensitivity analyses excluding BMI, hypertension, diabetes, and dyslipidemia from the covariable set yielded results largely consistent with the primary analysis (Supplementary Table S9), indicating that the observed associations were not substantially affected by potential overadjustment. These findings aligned with the primary analysis, indicating that our study results were robust.

## 4. Discussion

Our study represents the first investigation of the independent and joint associations between functional impairment and depression with advanced CKM syndrome among individuals of middle and advanced age, utilizing two large databases from different countries. After adjusting for sex, age, educational attainment, marital status, BMI, smoking status, drinking status, hypertension, diabetes, dyslipidemia, and total sleep time, it was found that functional impairment and depression were independently associated with advanced CKM syndrome, and the coexistence of both conditions was associated with an even greater predisposition to advanced CKM syndrome. These findings reveal the high-risk characteristics of individuals with dyslipidemia and clarify the independent and joint roles of functional impairment and depression in forecasting the likelihood of developing advanced CKM syndrome among individuals of middle and old age. Therefore, this study may help identify individuals with co-occurring functional impairment and depression as a group with higher odds of advanced CKM syndrome.

Functional status and psychological factors are crucial for CKM syndrome, as they can influence disease progression, lead to cardiovascular complications, and increase mortality. This may be related to dyslipidemia, metabolic disorders, and abnormalities in cardiac autonomic regulation caused by functional impairment [12,37], as well as compromised heart rate variability, systemic chronic inflammation, hypothalamic–pituitary–adrenal (HPA) axis dysregulation, and vascular endothelial dysfunction attributed to depression [38]. Driven by the rapidly aging population, functional impairment and depression are becoming increasingly prevalent among individuals of middle and old age [39,40]. Furthermore, functional impairment and depression often co-occur in older individuals [20,41,42]. This study found that the combination of functional impairment and depression was significantly associated with advanced CKM syndrome among individuals of middle and old age, and this association was stronger than that of either condition alone, which may be related to multiple mechanisms. First, functional impairment and depression can synergistically activate monocytes or macrophages, leading to the sustained and excessive production of systemic inflammatory cytokines like interleukin-6 (IL-6) and tumor necrosis factor-α (TNF-α), while chronic inflammation is a core pathological feature of CKM syndrome [43]. Second, the synergistic effects of functional impairment and depression can trigger elevated blood pressure, abnormal blood glucose levels, and insufficient renal perfusion by mediating the excessive activation of the HPA axis as well as elevated cortisol levels [44]. Furthermore, the HPA axis exhibits a detrimental interplay with inflammatory pathways, as pro-inflammatory cytokines can exacerbate the dysfunction of the HPA axis, while HPA axis activation further enhances the inflammatory response [45]. In addition to the aforementioned pathophysiological mechanisms, the combined effects of functional impairment and depression may also contribute to the initiation and development of advanced CKM syndrome through unhealthy lifestyles. This is because these two factors synergistically lead to reduced physical activity and poor dietary adherence, thereby exacerbating metabolic abnormalities such as obesity and insulin resistance [11,46]. In addition, they reduce patients’ medication adherence and disease self-management capabilities, ultimately leading to decompensation across multiple organ systems associated with CKM syndrome [47]. Therefore, functional impairment and depression may act synergistically through multiple pathways to accelerate the progression of CKM syndrome.

Our analysis revealed that depression moderated the association of functional impairment with advanced CKM syndrome in the NHANES sample but did not in the CHARLS cohort. Conversely, functional impairment moderated the association of depression with advanced CKM syndrome in the CHARLS cohort but did not in the NHANES sample. These differing results across the two datasets may be related to variations in the demographic characteristics of the populations (e.g., participants with advanced CKM syndrome in the NHANES sample were mostly older or obese). Multiple studies have shown that differences in the baseline characteristics of study populations, such as age, weight, and state of life, can directly or indirectly influence disease progression [48,49,50]. Beyond baseline demographic differences, significant disparities exist between Chinese and American populations in dietary structure, physical activity habits, perceptions of body weight, and awareness of mental health, which may lead to heterogeneity in the associations of depression and functional impairment with metabolic abnormalities. Furthermore, the U.S. healthcare system emphasizes individualized management of chronic diseases and screening for complications. However, older adults, obese individuals, and those with multiple comorbidities are more likely to experience concurrent depression and functional limitations, thereby accelerating the progression of CKM syndrome. In contrast, home care is more prevalent among older adults in China. Differences in disease self-management and social support models may lead to variations in the relative contributions and interaction patterns of functional impairment and depression.

Subgroup analyses showed that the interaction on the association of functional impairment and depression with advanced CKM syndrome did not differ significantly across most of the variables examined, with no consistent pattern observed between the two datasets. Nonetheless, several biological mechanisms may help explain the overall associations. Dyslipidemia can induce aggregation and phenotypic changes in macrophages, leading to the excessive secretion of pro-inflammatory cytokines (IL-6 and TNF-α) and systemic inflammatory response [51]. Dyslipidemia can also stimulate endothelial cells to produce multiple adhesion molecules (vascular cell adhesion molecule-1 and intercellular adhesion molecule-1) and chemokines (E-selectin), thereby causing dysfunction and structural changes in blood vessels [52]. Research evidence has indicated that both the accumulation and composition of lipids contribute to the pathophysiological process of lipotoxicity-related kidney injury by inducing inflammatory responses, oxidative stress, mitochondrial dysfunction, and apoptosis [53]. Functional impairment and depression can further exacerbate the systemic inflammation caused by dyslipidemia, aggravate vascular injury and renal function deterioration, and ultimately lead to the development of advanced CKM syndrome. Chronic drinking status not only leads to alcoholic fatty liver disease but also causes damage to renal tissue and cardiomyocytes by increasing oxidative stress and inducing mitochondrial dysfunction [54]. Sleep deprivation may exert multiple biological effects on the metabolic, endocrine, and immune systems as well as adverse effects on risk factors for CVD [55].

Through a cross-sectional analysis of the NHANES database and a cohort analysis of the CHARLS database, this study identified significant associations of functional impairment and depression with advanced CKM syndrome, revealing the systemic effects of functional status and psychological factors. However, our study has several limitations. First, despite adjustment for multiple covariables in the analysis, some unaccounted confounders, including dietary habits, nutritional status, and genetic susceptibility, may influence the study results. Second, the diagnosis of functional impairment and depression and the assessment of some covariables are based on data reported by participants, which may result in measurement errors and information bias in data collection. Additionally, NHANES and CHARLS employed different instruments to assess depression (PHQ-9 vs. CES-D-10) and functional impairment (different sets of BADL and IADL items). Although both instruments for each domain have been validated in their respective populations, measurement invariance across these instruments cannot be assumed. Third, the NHANES portion is cross-sectional, precluding temporal inference or causal conclusions, while the prospective design of CHARLS partially addresses temporal ordering by measuring baseline exposure (2011) before follow-up outcome (2015). Given that this is an observational study, we can only identify associations rather than establish causality, and the potential for reverse causality bias cannot be avoided. Finally, this study focused on multiplicative interaction through logistic regression, and additive interaction analyses (e.g., relative excess risk due to interaction [RERI], synergy index [SI]) were not performed. Future studies could employ additive interaction approaches to further elucidate the combined effects of functional impairment and depression on CKM syndrome. In addition, multicenter clinical studies employing standardized assessments are warranted to standardize the definitions of indicators and improve the identification of confounders. Furthermore, well-designed interventional trials are needed to determine whether integrated physical and mental health interventions can effectively delay the progression of CKM syndrome.

## 5. Conclusions

This study revealed that, in both the NHANES and CHARLS datasets, functional impairment and depression showed a strong positive association with advanced CKM syndrome. The consistency of this finding across two distinct populations, differing in geographic region, healthcare context, and study design, strengthens the evidence for an association between coexisting physical and mental health impairments and advanced CKM syndrome. Clinically, these findings highlight the need to increase attention to both physical and mental health, particularly through early psychological screening, functional assessment, and comprehensive risk management in this population.

## Figures and Tables

**Table 1:** Baseline characteristics of participants of NHANES.

Characteristics	Total (N = 3332)	Non-Advanced CKM (N = 1840)	Advanced CKM (N = 1492)	*p*-Value
Age, M (Q1, Q3)	66 (61, 74)	63 (59, 67)	73 (65, 80)	<0.001
Sex, *n* (%)				<0.001
Female	1727 (52%)	1078 (59%)	649 (43%)	
Male	1605 (48%)	762 (41%)	843 (57%)	
Marital status, *n* (%)				<0.001
Married/Living with partner	1935 (58%)	1107 (60%)	828 (55%)	
Widowed/Divorced/Separated	1147 (34%)	560 (30%)	587 (39%)	
Never married	250 (8%)	173 (10%)	77 (6%)	
Educational attainment, *n* (%)				<0.001
Less than high school	841 (25%)	425 (23%)	416 (28%)	
High school	804 (24%)	417 (23%)	387 (26%)	
More than high school	1687 (51%)	998 (54%)	689 (46%)	
BMI (kg/m^2^), M (Q1, Q3)	29 (25, 33)	28 (25, 33)	29 (26, 34)	<0.001
BMI group (kg/m^2^), *n* (%)				0.001
<24.9	792 (24%)	481 (26%)	311 (21%)	
≥25 to <30	1140 (34%)	622 (34%)	518 (35%)	
≥30	1400 (42%)	737 (40%)	663 (44%)	
Smoking status, *n* (%)				<0.001
No	1589 (48%)	1028 (56%)	561 (38%)	
Yes	1743 (52%)	812 (44%)	931 (62%)	
Drinking status, *n* (%)				0.929
No	888 (27%)	492 (27%)	396 (27%)	
Yes	2444 (73%)	1348 (73%)	1096 (73%)	
Total sleep time (h), *n* (%)				0.023
<7	934 (28%)	531 (29%)	403 (27%)	
≥7	2398 (72%)	1309 (71%)	1089 (73%)	
Hypertension, *n* (%)				<0.001
No	1056 (32%)	814 (44%)	242 (16%)	
Yes	2276 (68%)	1026 (56%)	1250 (84%)	
Diabetes, *n* (%)				<0.001
No	2201 (66%)	1464 (80%)	737 (49%)	
Yes	1131 (34%)	376 (20%)	755 (51%)	
Dyslipidemia, *n* (%)				<0.001
No	495 (15%)	316 (17%)	179 (12%)	
Yes	2837 (85%)	1524 (83%)	1313 (88%)	
Depression score, M (Q1, Q3)	2.0 (0.0, 5.0)	2.0 (0.0, 5.0)	2.0 (0.0, 6.0)	0.13
Depression, *n* (%)				0.174
No	2937 (88%)	1635 (89%)	1302 (87%)	
Yes	395 (12%)	205 (11%)	190 (13%)	
IADL, *n* (%)				<0.001
No	2260 (68%)	1309 (71%)	951 (64%)	
Yes	1072 (32%)	531 (29%)	541 (36%)	
BADL, *n* (%)				<0.001
No	2432 (73%)	1418 (77%)	1014 (68%)	
Yes	900 (27%)	422 (23%)	478 (32%)	
IADL and depression				<0.001
Non-IADL and non-depression	2122 (64%)	1233 (67%)	889 (60%)	
Non-IADL and depression	138 (4%)	76 (4%)	62 (4%)	
IADL and non-depression	815 (24%)	402 (22%)	413 (28%)	
IADL and depression	257 (8%)	129 (7%)	128 (8%)	
BADL and depression				<0.001
Non-BADL and non-depression	2263 (68%)	1317 (72%)	946 (63%)	
Non-BADL and depression	169 (5%)	101 (5%)	68 (5%)	
BADL and non-depression	674 (20%)	318 (17%)	356 (24%)	
BADL and depression	226 (7%)	104 (6%)	122 (8%)	

Abbreviations: CKM: cardiovascular–kidney–metabolic syndrome; BMI: body mass in dex; IADL: Instrumental activity of daily living; BADL: Basic activities of daily living; M: Median; Q1: The first quartile (25th percentile); Q3: The third quartile (75th percentile).

**Table 2:** Baseline characteristics of participants of CHARLS.

Characteristics	Total (N = 4631)	Non-Advanced CKM (N = 4384)	Advanced CKM (N = 247)	*p*-Value
Age, M (Q1, Q3)	58 (52, 64)	58 (52, 64)	60 (53, 66)	0.003
Sex, *n* (%)				<0.001
Female	2569 (55%)	2459 (56%)	110 (45%)	
Male	2062 (45%)	1925 (44%)	137 (55%)	
Marital status, *n* (%)				0.632
Married/Living with partner	4139 (89%)	3921 (89%)	218 (88%)	
Others	492 (11%)	463 (11%)	29 (12%)	
Educational attainment, *n* (%)				<0.001
High school and below	392 (8%)	370 (8%)	22 (9%)	
More than high school	4239 (92%)	4014 (92%)	225 (91%)	
BMI (kg/m^2^), M (Q1, Q3)	23.0 (20.9, 25.5)	23.0 (20.8, 25.4)	23.9 (21.7, 26.1)	<0.001
BMI group (kg/m^2^), *n* (%)				0.005
<24.9	3262 (70%)	3110 (71%)	152 (62%)	
≥25 to <28	854 (18%)	797 (18%)	57 (23%)	
≥28	510 (11%)	472 (11%)	38 (15%)	
Smoking status, *n* (%)				<0.001
No	1717 (37%)	1599 (36%)	118 (48%)	
Yes	2914 (63%)	2785 (64%)	129 (52%)	
Drinking status, *n* (%)				>0.999
No	3096 (67%)	2931 (67%)	165 (67%)	
Yes	1535 (33%)	1453 (33%)	82 (33%)	
Total sleep time (h), n (%)				0.023
<7	1938 (42%)	1830 (42%)	108 (44%)	
≥7	2693 (58%)	2554 (58%)	139 (56%)	
Hypertension, *n* (%)				<0.001
No	2939 (63%)	2840 (65%)	99 (40%)	
Yes	1692 (37%)	1544 (35%)	148 (60%)	
Diabetes, *n* (%)				<0.001
No	3965 (86%)	3773 (86%)	192 (78%)	
Yes	666 (14%)	611 (14%)	55 (22%)	
Dyslipidemia, *n* (%)				0.011
No	1493 (32%)	1432 (33%)	61 (25%)	
Yes	3138 (68%)	2952 (67%)	186 (75%)	
Depression score, M (Q1, Q3)	7 (3,12)	7 (3,12)	9 (3,14)	0.021
Depression, *n* (%)				0.001
No	2930 (63%)	2798 (64%)	132 (53%)	
Yes	1701 (37%)	1586 (36%)	115 (47%)	
IADL, *n* (%)				0.004
No	3797 (82%)	3612 (82%)	185 (75%)	
Yes	834 (18%)	772 (18%)	62 (25%)	
BADL, *n* (%)				0.003
No	3967 (86%)	3772 (86%)	195 (79%)	
Yes	664 (14%)	612 (14%)	52 (21%)	
IADL and depression				0.001
Non-IADL and non-depression	2625 (57%)	2511 (57%)	114 (46%)	
Non-IADL and depression	1172 (25%)	1101 (25%)	71 (29%)	
IADL and non-depression	305 (7%)	287 (7%)	18 (7%)	
IADL and depression	529 (11%)	485 (11%)	44 (18%)	
BADL and depression				<0.001
Non-BADL and non-depression	2698 (58%)	2581 (59%)	117 (47%)	
Non-BADL and depression	1269 (27%)	1191 (27%)	78 (32%)	
BADL and non-depression	232 (5%)	217 (5%)	15 (6%)	
BADL and depression	432 (10%)	395 (9%)	37 (15%)	

Notes: CKM: cardiovascular–kidney–metabolic syndrome; BMI: body mass index; IADL: Instrumental activity of daily living; BADL: Basic activities of daily living; M: Median; Q1: The first quartile (25th percentile); Q3: The third quartile (75th percentile).

**Table 3:** Individual and Joint Associations of ADL and Depression in NHANES.

	Model 1		Model 2		Model 3	
	OR (95% CI)	*p*	OR (95% CI)	*p*	OR (95% CI)	*p*
Depression						
No	Ref	Ref	Ref	Ref	Ref	Ref
Yes	3.930 (2.681, 5.760)	<0.001	3.457 (2.355, 5.073)	<0.001	3.306 (2.149, 5.086)	<0.001
IADL						
No	Ref	Ref	Ref	Ref	Ref	Ref
Yes	2.796 (2.050, 3.812)	<0.001	2.551 (1.886, 3.451)	<0.001	2.147 (1.520, 3.033)	<0.001
BADL						
No	Ref	Ref	Ref	Ref	Ref	Ref
Yes	2.912 (2.172, 3.904)	<0.001	2.694 (2.040, 3.559)	<0.001	2.120 (1.565, 2.872)	<0.001
IADL and depression						
Non-IADL and non-depression	Ref	Ref	Ref	Ref	Ref	Ref
Non-IADL and depression	3.604 (2.172, 5.981)	<0.001	3.273 (2.028, 5.281)	<0.001	3.205 (1.709, 6.007)	<0.001
IADL and non-depression	2.487 (1.774, 3.487)	<0.001	2.320 (1.673, 3.216)	<0.001	1.949 (1.376, 2.761)	<0.001
IADL and depression	6.294 (3.713, 10.670)	<0.001	5.503 (3.240, 9.347)	<0.001	4.843 (2.505, 9.366)	<0.001
BADL and depression						
Non-BADL and non-depression	Ref	Ref	Ref	Ref	Ref	Ref
Non-BADL and depression	3.901 (2.448, 6.216)	<0.001	3.579 (2.278, 5.623)	<0.001	3.414 (1.858, 6.273)	<0.001
BADL and non-depression	2.665 (1.915, 3.710)	<0.001	2.538 (1.848, 3.487)	<0.001	1.942 (1.395, 2.703)	<0.001
BADL and depression	5.940 (3.357, 10.510)	<0.001	5.131 (2.894, 9.097)	<0.001	4.394 (2.281, 8.464)	<0.001

Notes: Model 1: adjusted age and sex; Model 2: adjusted educational attainment and marital status on Model 1; Model 3: adjusted BMI, smoking status, drinking status, hypertension, diabetes, dyslipidemia and total sleep time based on Model 2. Abbreviations: OR: Odds ratio; CI: Confidence interval; Ref: Reference; IADL: Instrumental activity of daily living; BADL: Basic activities of daily living.

**Table 4:** Individual and Joint Associations of ADL and Depression in CHARLS.

	Model 1		Model 2		Model 3	
	OR (95% CI)	*p*	OR (95% CI)	*p*	OR (95% CI)	*p*
Depression						
No	Ref	Ref	Ref	Ref	Ref	Ref
Yes	1.628 (1.252, 2.115)	<0.001	1.641 (1.260, 2.136)	<0.001	1.745 (1.324, 2.298)	<0.001
IADL						
No	Ref	Ref	Ref	Ref	Ref	Ref
Yes	1.599 (1.169, 2.161)	0.003	1.606 (1.173, 2.172)	0.003	1.586 (1.147, 2.166)	0.004
BADL						
No	Ref	Ref	Ref	Ref	Ref	Ref
Yes	1.624 (1.164, 2.232)	0.003	1.632 (1.168, 2.245)	0.003	1.585 (1.124, 2.200)	0.007
IADL and depression						
Non-IADL and non-depression	Ref	Ref	Ref	Ref	Ref	Ref
Non-IADL and depression	1.510 (1.106, 2.048)	0.009	1.523 (1.114, 2.070)	0.008	1.640 (1.185, 2.259)	0.003
IADL and non-depression	1.387 (0.802, 2.269)	0.215	1.396 (0.807, 2.287)	0.206	1.393 (0.796, 2.306)	0.22
IADL and depression	2.153 (1.470, 3.104)	<0.001	2.178 (1.484, 3.147)	<0.001	2.259 (1.517, 3.319)	<0.001
BADL and depression						
Non-BADL and non-depression	Ref	Ref	Ref	Ref	Ref	Ref
Non-BADL and depression	1.551 (1.148, 2.085)	0.004	1.566 (1.157, 2.110)	0.003	1.650 (1.206, 2.247)	0.002
BADL and non-depression	1.511 (0.831, 2.566)	0.149	1.528 (0.839, 2.599)	0.139	1.377 (0.747, 2.373)	0.275
BADL and depression	2.140 (1.428, 3.142)	<0.001	2.161 (1.440, 3.178)	<0.001	2.287 (1.501, 3.419)	<0.001

Notes: Model 1: adjusted age and sex; Model 2: adjusted educational attainment and marital status on Model 1; Model 3: adjusted BMI, smoking status, drinking status, hypertension, diabetes, dyslipidemia and total sleep time based on Model 2. Abbreviations: OR: Odds ratio; CI: Confidence interval; Ref: Reference; IADL: Instrumental activity of daily living; BADL: Basic activities of daily living.

## Data Availability

The data presented in this study are available on the China Health and Retirement Longitudinal Study (CHARLS) website at http://charls.pku.edu.cn/ (accessed on 10 July 2025), and on the National Health and Nutrition Examination Survey (NHANES) website at https://wwwn.cdc.gov/nchs/nhanes/ (accessed on 10 July 2025).

## Supplementary Materials

The following supporting information can be downloaded at: https://www.mdpi.com/article/10.3390/healthcare14142043/s1, Table S1: CKM Syndrome Staging: Criteria and Thresholds; Table S2: Multicollinearity test of variables at IADL and depression; Table S3: Multicollinearity test of variables at BADL and depression; Table S4: The moderating effect of depression on IADL; Table S5: The moderating effect of depression on BADL; Table S6: The moderating effect of IADL on depression; Table S7: The moderating effect of BADL on depression; Table S8: Sensitivity analysis results; Table S9: The association between depression leading to functional impairment and late-stage CKM (after excluding hypertension, hyperlipidemia, diabetes, and BMI).

## Abbreviations

CKM, Cardiovascular-kidney-metabolic; CKD, chronic kidney disease; CVD, cardiovascular disease; ADL, activities of daily living; NHANES, National Health and Nutrition Examination Survey; CHARLS, China Health and Retirement Longitudinal Study; SD, Standard Deviation; IQR, interquartile range; VIF, variance inflation factor.
