# Supplementary material for: Joint Association of Functional Impairment and Depression with Advanced Cardiovascular–Kidney–Metabolic Syndrome: A Cross-Sectional and Cohort Study Using Two Large Datasets
Source: Healthcare (Basel). 2026 Jul 8;14(14):2043. doi: 10.3390/healthcare14142043 (PMC13411224; doi:10.3390/healthcare14142043)
Supplement: Supplementary file 1 [file healthcare-14-02043-s001.zip › healthcare-4321506-supplementary.pdf]

Supplementary Materials

Table S1 CKM Syndrome Staging: Criteria and Thresholds.

CKM Stage	Definition	Criteria
Stage 0	Normal metabolic markers	(1) BMI ethnicity-specific threshold: Asian < 23 kg/m²; non-Asian < 25 kg/m²; (2) Waist circumference ethnicity- and sex-specific threshold: Asian men < 90 cm, Asian women < 80 cm; non-Asian men < 102 cm, non-Asian women < 88 cm
Stage 1	Excess adiposity or prediabetes	(1) BMI ≥ above threshold; WC ≥ above threshold; (2) HbA1c 5.7%–6.5%; fasting glucose 100–125 mg/dL
Stage 2	Metabolic risk factors	(1) Metabolic syndrome: (i) Elevated waist circumference: Asian men ⩾ 90 cm, Asian women ⩾ 80 cm; non-Asian men ⩾ 102 cm, non-Asian women ⩾ 88 cm; (ii) Reduced HDL: < 40 mg/dL (men), < 50 mg/dL (women); (iii) Elevated triglycerides: ⩾ 150 mg/dL; (iv) Elevated blood pressure: ⩾ 130/80 mmHg;

		(v) Elevated glucose or HbA1c: fasting glucose ⩾ 100 mg/dL or HbA1c ⩾ 5.7%
		Metabolic syndrome was defined as the presence of ⩾ 3 of the above 5 criteria;
		(2) Moderately decreased eGFR 30–59 mL/min/1.73m²
Stage 3	Very high-risk CKD or very high CVD risk	(1) Severely decreased eGFR < 30 mL/min/1.73m²;
		(2) High 10-year CVD risk: Framingham risk score ≥ 21.6% (men), ≥ 21.5% (women)
Stage 4	Established CVD	Self-reported history of heart disease or stroke

Table S2 Multicollinearity test of variables at IADL and depression.

	NHANES						CHARLS					
	Model1		Model2		Model3		Model1		Model2		Model3	
	OR(95%CI)	P	OR(95%CI)	P	OR(95%CI)	P	OR(95%CI)	P	OR(95%CI)	P	OR(95%CI)	P
Non-depression												
Non-IADL	Ref	Ref	Ref	Ref	Ref	Ref	Ref	Ref	Ref	Ref	Ref	Ref
IADL	2.586(1.843, 3.630)	<0.001	2.418(1.740, 3.361)	<0.001	2.035(1.424, 2.907)	<0.001	1.411(0.810, 2.325)	0.198	1.427(0.818,2.356)	0.185	1.485(0.840, 2.486)	0.151
Depression												
Non-IADL	Ref	Ref	Ref	Ref	Ref	Ref	Ref	Ref	Ref	Ref	Ref	Ref
IADL	1.484(0.803, 2.741)	0.202	1.395(0.765, 2.543)	0.269	1.327(0.762, 2.309)	0.307	1.399(0.933,2.079)	0.1	1.378(0.917, 2.051)	0.117	1.305(0.858, 1.965)	0.207

Notes: Model1: adjusted age and sex; Model2: adjusted educational attainment and marital status on model1; Model3: adjusted BMI, smoking status, drinking status,

hypertension, diabetes, dyslipidemia and total sleep time based on model2

Abbreviations: OR: Odds ratio; CI: Confidence interval; Ref: Reference; IADL: Instrumental activity of daily living

Table S3 Multicollinearity test of variables at BADL and depression.

	NHANES			CHARLS		
Variable	GVIF	Df	GVIF^(1/(2*Df))	GVIF	Df	GVIF^(1/(2*Df))
BADL and depression	1.189	3	1.029	1.634	3	1.026
Age	1.510	1	1.229	1.226	1	1.107
Sex	1.211	1	1.101	2.376	1	1.541
Education level	1.054	2	1.013	1.063	1	1.031
Marital status	1.106	2	1.026	1.093	1	1.045
BMI	1.139	2	1.033	1.190	2	1.044
Smoking status	1.318	1	1.148	2.123	1	1.457
Drinking status	1.212	1	1.101	1.294	1	1.138
Total sleep time	1.029	1	1.015	1.080	1	1.039
Hypertension	1.065	1	1.032	1.121	1	1.059
Diabetes	1.190	1	1.091	1.030	1	1.015
Dyslipidemia	1.025	1	1.013	1.090	1	1.044

Notes: BMI: body mass index; BADL: Basic activities of daily living; GVIF: Generalized variance inflation factor; Df: Degrees of freedom

Table S4 The moderating effect of depression on IADL.

	NHANES						CHARLS					
	Model1		Model2		Model3		Model1		Model2		Model3	
	OR(95%CI)	P	OR(95%CI)	P	OR(95%CI)	P	OR(95%CI)	P	OR(95%CI)	P	OR(95%CI)	P
Non-depression												
Non-IADL	Ref	Ref	Ref	Ref	Ref	Ref	Ref	Ref	Ref	Ref	Ref	Ref
IADL	2.586(1.843, 3.630)	<0.001	2.418(1.740, 3.361)	<0.001	2.035(1.424, 2.907)	<0.001	1.411(0.810, 2.325)	0.198	1.427(0.818,2.356)	0.185	1.485(0.840, 2.486)	0.151
Depression												
Non-IADL	Ref	Ref	Ref	Ref	Ref	Ref	Ref	Ref	Ref	Ref	Ref	Ref
IADL	1.484(0.803, 2.741)	0.202	1.395(0.765, 2.543)	0.269	1.327(0.762, 2.309)	0.307	1.399(0.933,2.079)	0.1	1.378(0.917, 2.051)	0.117	1.305(0.858, 1.965)	0.207

Notes: Model1: adjusted age and sex; Model2: adjusted educational attainment and marital status on model1; Model3: adjusted BMI, smoking status, drinking status,

hypertension, diabetes, dyslipidemia and total sleep time based on model2

Abbreviations: OR: Odds ratio; CI: Confidence interval; Ref: Reference; IADL: Instrumental activity of daily living

Table S5 The moderating effect of depression on BADL.

	NHANES						CHARLS					
	Model1		Model2		Model3		Model1		Model2		Model3	
	OR(95%CI)	P	OR(95%CI)	P	OR(95%CI)	P	OR(95%CI)	P	OR(95%CI)	P	OR(95%CI)	P
Non-depression												
Non-BADL	Ref	Ref	Ref	Ref	Ref	Ref	Ref	Ref	Ref	Ref	Ref	Ref
BADL	2.763(1.964, 3.886)	<0.001	2.637(1.904, 3.651)	<0.001	2.024(1.424, 2.877)	<0.001	1.538(0.841, 2.629)	0.136	1.576(0.860, 2.701)	0.117	1.402(0.754, 2.441)	0.257
Depression												
Non-BADL	Ref	Ref	Ref	Ref	Ref	Ref	Ref	Ref	Ref	Ref	Ref	Ref

BADL	1.389(0.768, 2.511)	0.271	1.275(0.715, 2.274)	0.402	1.252(0.691, 2.267)	0.448	1.367(0.894, 2.057)	0.14	1.349(0.882, 2.032)	0.158	1.358(0.878, 2.069)	0.161

Notes: Model1: adjusted age and sex; Model2: adjusted educational attainment and marital status on model1; Model3: adjusted BMI, smoking status, drinking status, hypertension, diabetes, dyslipidemia and total sleep time based on model2

Abbreviations: OR: Odds ratio; CI: Confidence interval; Ref: Reference; BADL: Basic activities of daily living

Table S6 The moderating effect of IADL on depression.

	NHANES						CHARLS					
	Model1		Model2		Model3		Model1		Model2		Model3	
	OR(95%CI)	P	OR(95%CI)	P	OR(95%CI)	P	OR(95%CI)	P	OR(95%CI)	P	OR(95%CI)	P
Non-IADL												
Non-depression	Ref	Ref	Ref	Ref	Ref	Ref	Ref	Ref	Ref	Ref	Ref	Ref
Depression	4.160(2.337, 7.403)	<0.001	3.776(2.220, 6.424)	<0.001	3.954(1.935, 8.078)	<0.001	1.503(1.100, 2.041)	0.01	1.496(1.093, 2.037)	0.011	1.631(1.178, 2.247)	0.003
IADL												
Non-depression	Ref	Ref	Ref	Ref	Ref	Ref	Ref	Ref	Ref	Ref	Ref	Ref
Depression	2.161(1.320, 3.538)	0.003	2.015(1.248, 3.256)	0.005	1.971(1.204, 3.225)	0.008	1.569(0.902, 2.839)	0.122	1.612(0.925, 2.923)	0.102	1.630(0.898,3.069)	0.118

Notes: Model1: adjusted age and sex; Model2: adjusted educational attainment and marital status on model1; Model3: adjusted BMI, smoking status, drinking status, hypertension, diabetes, dyslipidemia and total sleep time based on model2

Abbreviations: OR: Odds ratio; CI: Confidence interval; Ref: Reference; IADL: Instrumental activity of daily living

Table S7 The moderating effect of BADL on depression.

	NHANES						CHARLS					
	Model1		Model2		Model3		Model1		Model2		Model3	
	OR(95%CI)	P	OR(95%CI)	P	OR(95%CI)	P	OR(95%CI)	P	OR(95%CI)	P	OR(95%CI)	P
Non-BADL												
Non-depression	Ref	Ref	Ref	Ref	Ref	Ref	Ref	Ref	Ref	Ref	Ref	Ref
Depression	4.229(2.542, 7.036)	<0.001	3.906(2.375, 6.423)	<0.001	3.769(1.966, 7.224)	<0.001	1.567(1.159, 2.109)	0.003	1.563(1.153, 2.109)	0.004	1.675(1.224, 2.285)	0.001
BADL												
Non-depression	Ref	Ref	Ref	Ref	Ref	Ref	Ref	Ref	Ref	Ref	Ref	Ref
Depression	1.960(1.137,3.379)	0.016	1.805(1.043, 3.124)	0.035	1.996(1.171, 3.404)	0.012	1.386(0.755, 2.667)	0.308	1.395(0.760, 2.683)	0.298	1.559(0.807, 3.152)	0.199

Notes: Model1: adjusted age and sex; Model2: adjusted educational attainment and marital status on model1; Model3: adjusted BMI, smoking status, drinking status, hypertension, diabetes, dyslipidemia and total sleep time based on model2

Abbreviations: OR: Odds ratio; CI: Confidence interval; Ref: Reference; BADL: Basic activities of daily living

Table S8 Sensitivity analysis results.

	Model1		Model2		Model3	
	OR(95%CI)	P	OR(95%CI)	P	OR(95%CI)	P
NHANES						
Depression						
No	Ref	Ref	Ref	Ref	Ref	Ref
Yes	3.854(2.635, 5.637)	<0.001	3.394(2.319, 4.968)	<0.001	3.197(2.069, 4.938)	<0.001
IADL						
No	Ref	Ref	Ref	Ref	Ref	Ref
Yes	2.662(1.944, 3.645)	<0.001	2.431(1.787, 3.306)	<0.001	2.020(1.413, 2.887)	<0.001
BADL						
No	Ref	Ref	Ref	Ref	Ref	Ref
Yes	2.784(2.078, 3.731)	<0.001	2.557(1.952, 3.402)	<0.001	2.011(1.483, 2.727)	<0.001
IADL and depression						
Non-IADL and non-depression	Ref	Ref	Ref	Ref	Ref	Ref

Non-IADL and depression	3.713(2.210, 6.238)	<0.001	3.372(2.063, 5.511)	<0.001	3.338(1.747, 6.378)	<0.001
IADL and non-depression	2.386(1.695, 3.358)	<0.001	2.227(1.597, 3.107)	<0.001	1.862(1.299, 2.670)	0.001
IADL and depression	5.847(3.445, 9.924)	<0.001	5.115(3.010, 8.691)	<0.001	4.336(2.207, 8.519)	<0.001
BADL and depression						
Non-BADL and non-depression	Ref	Ref	Ref	Ref	Ref	Ref
Non-BADL and depression	3.992(2.477, 6.432)	<0.001	3.670(2.309, 5.831)	<0.001	3.582(1.921, 6.680)	<0.001
BADL and non-depression	2.574(1.852, 3.577)	<0.001	2.453(1.786, 3.370)	<0.001	1.889(1.356, 2.633)	<0.001
BADL and depression	5.504(3.102, 9.766)	<0.001	4.748(2.682, 8.406)	<0.001	3.893(2.004, 7.562)	<0.001
CHARLS						
Depression						
No	Ref	Ref	Ref	Ref	Ref	Ref
Yes	1.629(1.252, 2.117)	<0.001	1.642(1.260, 2.139)	<0.001	1.745(1.323, 2.299)	<0.001

IADL						
No	Ref	Ref	Ref	Ref	Ref	Ref
Yes	1.587(1.157, 2.148)	0.003	1.594(1.162, 2.160)	0.003	1.575(1.137, 2.156)	0.005
BADL						
No	Ref	Ref	Ref	Ref	Ref	Ref
Yes	1.645(1.178, 2.262)	0.003	1.653(1.183, 2.275)	0.003	1.606(1.139, 2.230)	0.006
IADL and depression						
Non-IADL and non-depression	Ref	Ref	Ref	Ref	Ref	Ref
Non-IADL and depression	1.517(1.111,2.058)	0.008	1.530(1.119, 2.080)	0.007	1.647(1.190, 2.268)	0.002
IADL and non-depression	1.383(0.799, 2.264)	0.219	1.393(0.805, 2.281)	0.21	1.394(0.797, 2.307)	0.218
IADL and depression	2.143(1.458, 3.100)	<0.001	2.167(1.472, 3.141)	<0.001	2.247(1.504, 3.309)	<0.001
BADL and depression						
Non-BADL and non-depression	Ref	Ref	Ref	Ref	Ref	Ref

Non-BADL and depression	1.540(1.138, 2.072)	0.005	1.554(1.147, 2.097)	0.004	1.638(1.196, 2.233)	0.002
BADL and non-depression	1.500(0.825, 2.548)	0.156	1.517(0.833, 2.581)	0.146	1.373(0.745, 2.365)	0.28
BADL and depression	2.180(1.454, 3.204)	<0.001	2.202(1.466, 3.241)	<0.001	2.329(1.528, 3.486)	<0.001

Notes: Model 1: adjusted age and sex; Model 2: adjusted educational attainment and marital status on Model 1; Model 3: adjusted BMI, smoking status, drinking status, hypertension, diabetes, dyslipidemia and total sleep time based on Model 2.

Abbreviations: OR: Odds ratio; CI: Confidence interval; Ref: Reference; IADL: Instrumental activity of daily living; BADL: Basic activities of daily living.

Table S9 The association between depression leading to functional impairment and late-stage CKM (after excluding hypertension, hyperlipidemia, diabetes, and BMI).

	NHANES		CHARLS	
	OR(95%CI)	P	OR(95%CI)	P
Depression				
No	Ref	Ref	Ref	Ref
Yes	3.035(2.126, 4.334)	<0.001	1.629(1.242, 2.134)	<0.001
IADL				
No	Ref	Ref	Ref	Ref
Yes	2.577(2.063, 3.219)	<0.001	1.599(1.166, 2.167)	0.003
BADL				
No	Ref	Ref	Ref	Ref
Yes	2.800(2.203, 3.557)	<0.001	1.630(1.164, 2.248)	0.004
IADL and depression				
Non-IADL and non-depression	Ref	Ref	Ref	Ref
Non-IADL and depression	2.431(1.394, 4.239)	0.002	1.515(1.102, 2.070)	0.01
IADL and non-depression	2.324(1.828, 2.956)	<0.001	1.409(0.813, 2.311)	0.196

IADL and depression	5.637(3.555, 8.940)	<0.001	2.168(1.469, 3.154)	<0.001
BADL and depression				
Non-BADL and non-depression	Ref	Ref	Ref	Ref
Non-BADL and depression	2.711(1.682, 4.371)	<0.001	1.562(1.148, 2.116)	0.004
BADL and non-depression	2.600(2.015, 3.354)	<0.001	1.521(0.834, 2.590)	0.144
BADL and depression	5.389(3.237, 8.973)	<0.001	2.124(1.407, 3.146)	<0.001

Notes: this model adjusted age, gender, education level, marrital status, smoking status, drinking status and total sleep time

Abbreviations: OR: Odds ratio; CI: Confidence interval; Ref: Reference; IADL: Instrumental activity of daily living; BADL: Basic activities of daily living
